# In vivo assessment of pediatric kidney function using multi-parametric and multi-nuclear functional magnetic resonance imaging: challenges, perspectives, and clinical applications

**DOI:** 10.1007/s00467-024-06560-w

**Published:** 2024-11-18

**Authors:** Aurélie De Mul, Maxime Schleef, Guido Filler, Christopher McIntyre, Sandrine Lemoine

**Affiliations:** 1 Service de Néphrologie Et d’exploration Fonctionnelle Rénale, Hôpital Édouard-Herriot, Hospices Civils de Lyon, Lyon, France; 2 Department of Paediatrics (Division of Nephrology) and Medicine (Division of Nephrology), Western University, and London Health Sciences Centre, London, ON Canada; 3 The Lilibeth Caberto Kidney Clinical Research Unit, London Health Sciences Centre, London, ON Canada; 4 Department of Biophysics, Western University, and London Health Sciences Centre, London, ON Canada; 5 Université, Lyon 1, Lyon, France; 6 Centre de Référence Des Maladies Rares du Calcium Et du Phosphore, Centre de Référence Des Maladies Rénales Rares, Filières de Santé Maladies Rares OSCAR, ORKID Et ERKNet, Lyon, France

**Keywords:** Multi-parametric MRI, Multi-nuclear MRI, ^23^Na MRI, ^31^P magnetic resonance imaging spectroscopy, BOLD, DWI, Kidney physiology

## Abstract

The conventional methods for assessing kidney function, such as glomerular filtration rate and microalbuminuria, provide only partial insight into kidney function. Multi-parametric and multi-nuclear functional resonance magnetic imaging (MRI) techniques are innovative approaches to unraveling kidney physiology. Multi-parametric MRI includes various sequences to evaluate kidney perfusion, tissue oxygenation, and microstructure characterization, including fibrosis—a key pathological event in acute and chronic kidney disease and in transplant patients—without the need for invasive kidney biopsy. Multi-nuclear MRI detects nuclei other than protons. ^23^Na MRI enables visualization of the corticomedullary gradient and assessment of tissue sodium storage, which can be particularly relevant for personalized medicine in salt-wasting tubular disorders. Meanwhile, ^31^P-MRS measures intracellular phosphate and ATP variations, providing insights into oxidative metabolism in the muscle during exercise and recovery. This technique can be useful for detecting subclinical ischemia in chronic kidney disease and in tubulopathies with kidney phosphate wasting. These techniques are non-invasive and do not involve radiation exposure, making them especially suitable for longitudinal and serial assessments. They enable in vivo evaluation of kidney function on a whole-organ basis within a short acquisition time and with the ability to distinguish between medullary and cortical compartments. Therefore, they offer considerable potential for pediatric patients. In this review, we provide a brief overview of the main imaging techniques, summarize available literature data on both adult and pediatric populations, and examine the perspectives and challenges associated with multi-parametric and multi-nuclear MRI.

**Graphical Abstract:**

**Figure:**
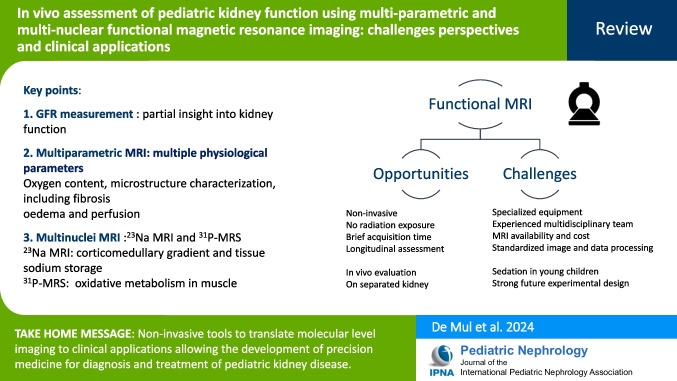
A higher resolution version of the Graphical abstract is available as Supplementary information

**Supplementary Information:**

The online version contains supplementary material available at 10.1007/s00467-024-06560-w.

## Introduction

Chronic kidney disease (CKD) represents a significant global health concern, affecting up to 10% of the adult population [1]. However, comprehensive epidemiological data on pediatric CKD are scarce, leading to an underestimation of its prevalence and incidence [2]. Despite this, pediatric CKD patients face significantly higher mortality rates—30 to 60 times greater than their healthy counterparts—alongside substantial morbidity, impacts on quality of life, and healthcare resource utilization [3]. The current definition of CKD, as proposed by the Kidney Disease: Improving Global Outcomes (KDIGO) guidelines, is primarily based on glomerular filtration rate (GFR) and albuminuria [4]. GFR can be estimated (eGFR) by different formulae using serum creatinine associated in the pediatric population with several limits [5]. It can also be assessed using exogenous markers like iohexol clearance, but these techniques are not widely accessible and require a significant amount of time and resources.

Moreover, GFR is only a partial surrogate for kidney function, as it fails to account for crucial aspects of kidney physiology such as medulla function, kidney perfusion, oxidative metabolism, and reabsorption function [5]. Kidney physiology is highly intricate. Despite being the most highly perfused organ, receiving 20 to 25% of cardiac output, the kidney exhibits a low oxygen extraction rate [6]. However, under pathological conditions, particularly in the medulla, susceptibility to hypoxia poses a significant problem, as it marks a critical final pathogenic event associated with CKD [7]. Medullary hypoxia can arise from the substantial metabolic demand and oxygen consumption due to tubular sodium reabsorption. Additionally, tissue perfusion in the kidney medulla is lower than in the cortex, with only 10% of blood reaching the medulla. Moreover, diffusive oxygen shunting occurs in vasculature bundles organized in a countercurrent arrangement. These factors collectively limit oxygen delivery to the medulla [8]. Hypoxic events promote fibrotic pathological processes, initiating a vicious circle where hyperfiltration occurs in remaining functional nephrons, increasing tubular reabsorptive workload and exacerbating hypoxia [9].

Consequences in kidney perfusion changes on tissue oxygenation cannot be reliably predicted through assessment of GFR, and there is no current physiological test to measure perfusion in clinical practice since para-amino-hippurate clearance is no longer available. Furthermore, fibrosis, a key predictor of declining kidney function, cannot be evaluated with GFR because of hyperfiltration of remaining glomeruli [9]. It requires a kidney biopsy to be estimated. Kidney biopsy is an invasive procedure, challenging to perform, almost never repeated in patients, with sampling bias [10]. Therefore, there is a crucial need for techniques capable of quantitatively assessing kidney tissue perfusion, oxygenation, and fibrosis in vivo on a whole-organ basis, enabling distinct evaluations for the left and right kidneys, as well as separate assessments of the cortex and medulla compartments [11]. In this context, this review addresses the description and clinical applications of kidney multi-parametric and multi-nuclear functional magnetic resonance imaging (MRI): a set of innovative tools for unraveling kidney physiology in the pediatric population.

## Multi-parametric magnetic resonance imaging

**Fig. 1:**
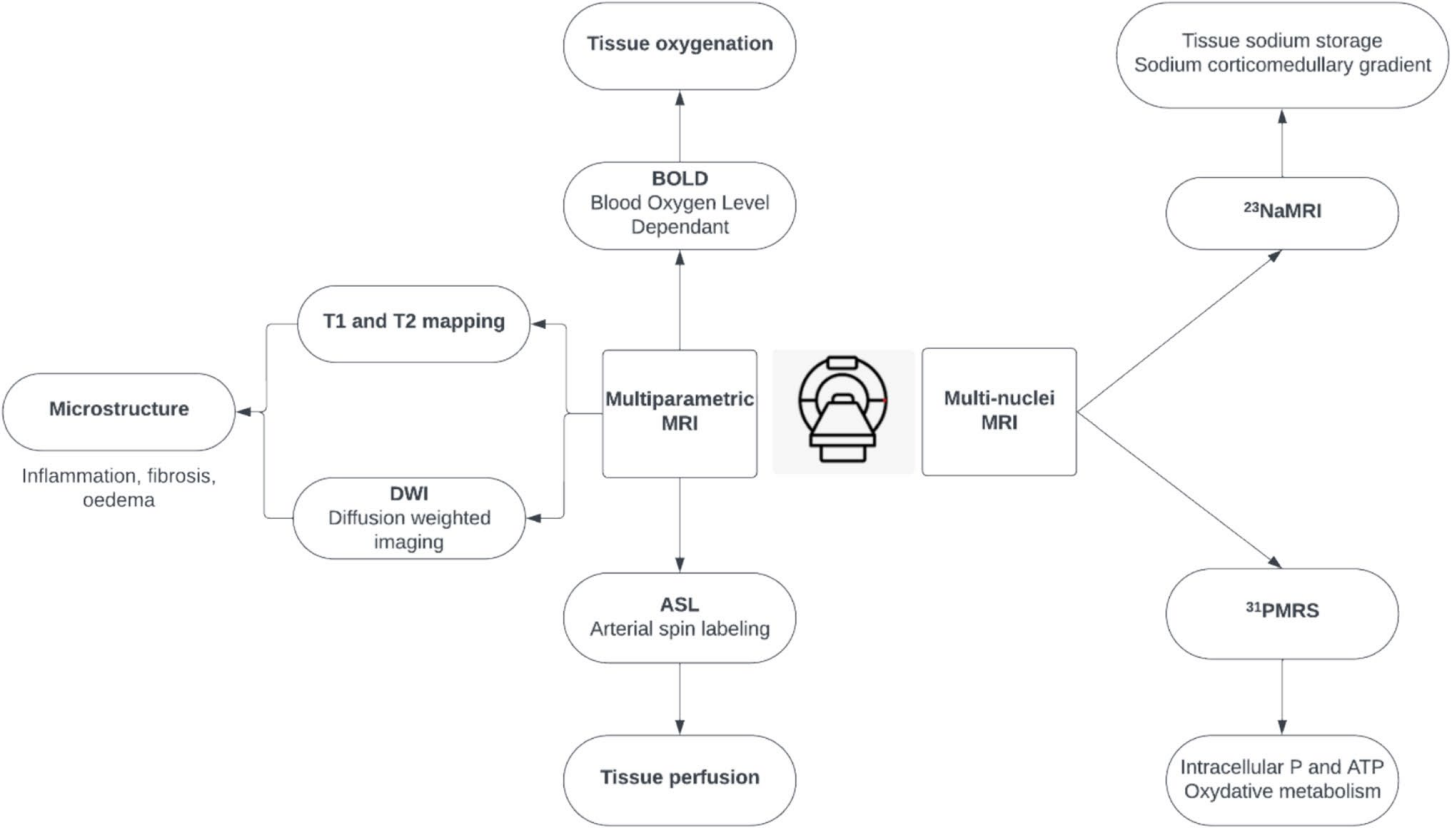
Overview of common multi-parametric and multi-nuclear resonance magnetic imaging techniques

Multi-parametric MRI involves different functional sequences that can be combined with conventional MRI high-resolution structural and anatomical images. These techniques enable the acquisition of functional parameters encompassing kidney perfusion, tissue oxygenation, or microstructure characterization (Fig. 1) [11]. We present an overview of the most commonly used multi-parametric MRI techniques in the field of nephrology. Other techniques, such as dynamic contrast-enhanced MRI, diffusion tensor imaging, and magnetic resonance elastography, also exist, and the range of MRI techniques continues to expand [12]. The aim of this review is to enhance the clinician’s understanding of these different MRI techniques. For more detailed physics concepts and technical information beyond the scope of this review, we refer readers to additional references [12, 13].

### The BOLD (blood oxygen level dependent) MRI sequence

**Fig. 2:**
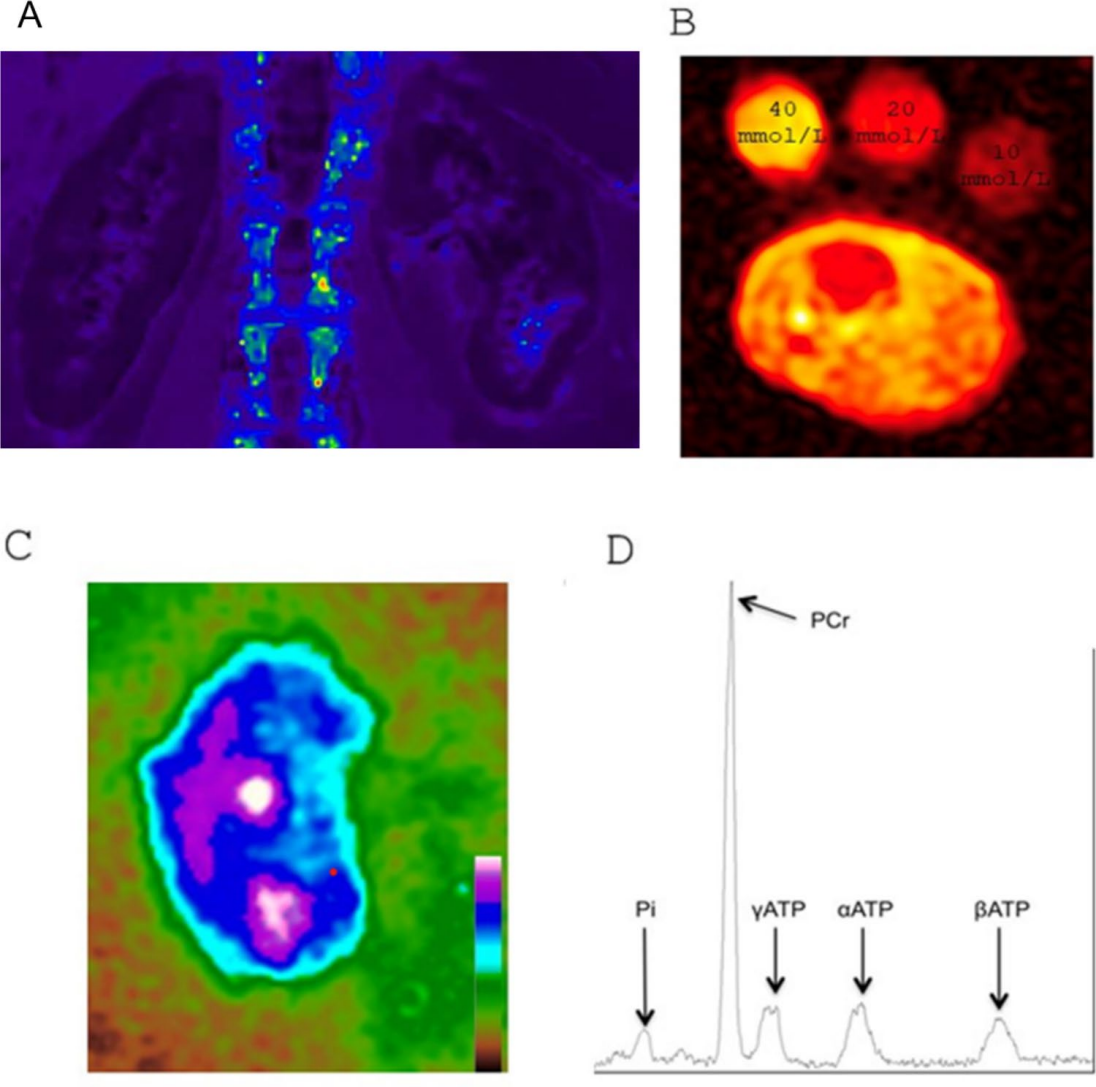
**A** Images of the kidneys acquired via BOLD-MRI in a healthy subject. **B** ^23^Na MRI of the leg in a healthy child. [Na^+^] measurement was possible by linear trend analysis, using three calibration vials containing increasing concentrations of NaCl solution. Tissue [Na^+^] is displayed as heat map, with greater signal intensity proportional to tissue [Na^+^]. **C** ^23^Na MRI along the kidney long axis in a healthy volunteer. **D** The acquired ^31^ P-MRS spectra showing phosphate (Pi), phospho-creatine (PCr), α, β-, and γ-ATP resonance peaks. The peak areas can be used to calculate concentrations, and the relative peak positions of Pi and PCr can be used to calculate the intracellular pH

is the most commonly used technique (Fig. 2A) to provide information on changes in kidney oxygenation or changes in the microstructure of the capillary bed. This is achieved by utilizing deoxyhemoglobin, which is paramagnetic and shortens the transverse relaxation constant T2* (ms), the inverse of the relaxation rate *R*2* (1/s). However, the BOLD signal is also affected by scanner-related factors such as motion artifacts, as well as physiological factors such as hematocrit, hydration status, and dietary sodium [14].

### T1 and T2 mapping

allows tissue characterization. Tissue alterations in water content such as inflammation, fibrosis, or edema modify the measurement of the longitudinal and transverse relaxation times. Distinguishable cortico-medullary differentiation (CMD) is viewable due to higher water mobility in the medulla [15].

### Diffusion-weighted imaging (DWI)

investigates random Brownian motion of water molecules to evaluate kidney microstructure modification, such as kidney fibrosis, inflammation, and edema [16]. Apparent diffusion coefficient (ADC) maps are classically created to visualize and measure water diffusion within tissues but also within blood microcirculation and tubules. The intravoxel incoherent motion (IVIM) model was created to better reflect physiological processes by separating true diffusion within tissues from other types of water movements.

### Arterial spin labeling (ASL)

is an experimental and not validated technique that enables the measurement of cortical and medullary perfusion without the need for exogenous contrast agents [17]. By utilizing blood water molecules as endogenous tracers magnetically labeled through radiofrequency pulses, kidney perfusion can be quantified leading to the creation of a quantitative perfusion map.

These sequences can be combined within a single MRI session. These techniques have been studied in research across various populations but have not yet been translated into clinical practice. In the adult population, correlations have been established between multi-parametric MRI and GFR in various clinical conditions such as CKD, kidney transplantation, patients with heart failure, and different kidney pathologies [18–21]. In patients with CKD, low cortical oxygenation has been suggested as an independent predictor of kidney function decline [22]. Moreover, these techniques may be used as surrogate markers for interstitial fibrosis in patients with CKD and transplant patients showing correlations with histological score [18, 20, 23, 24]. Due to limited patient numbers, studies in transplant patients yield conflicting results in distinguishing rejection from other dysfunctions or in identifying delayed graft function [24–28]. Nevertheless, their non-invasiveness makes them potentially useful in selecting patients for kidney biopsy. It can also help identify early stages of diabetic nephropathy, correlated with the extent of albuminuria [29]. These techniques can assess reduced cortical oxygenation in severe arterial stenosis, but their ability to predict kidney response to revascularization is uncertain [30–32]. It has sensitivity in detecting acute pyelonephritis [33]. While results are contradictory regarding obstruction detection, its utility for guiding decisions between watchful waiting and surgical intervention is still being studied [34].

### Pediatric considerations

Few studies, involving a small number of patients, have investigated the application of multi-parametric functional MRI in the pediatric population. These data are summarized in Table 1.

**Table 1:** Pediatric literature on the use of multi-parametric and multi-nuclear renal MRI

First author (year)	Population	Findings
**Multi-parametric MRI**		
*CKD*		
Liang P (2021) [35]	74 children (CKD stage 1–3, 51; CKD stage 4–5, 12; HC, 11)	BOLD and DWI MRI associated with eGFR, Cystatin C, and SCr levels
Dillman JR (2022) [36]	32 patients from 12 to 23 years of age (CKD, 12; HC, 20)	Cortical T1 values and whole kidney ADC correlated with eGFR and Cystatin C DWI ADC correlated with cortical histologic inflammation
Luo F (2020) [37]	43 children (CKD stage 1–3, 21; CKD stage 4–5: 16; HC, 6)	R2* values (BOLD) correlated with eGFR and SCr levels
*Kidney transplantation*		
Radovic T (2022) [38]	18 patients (median age 16 years (5–29); median time interval between transplantation and MRI: 74 months (2–187))	Mean cRBF (ASL-RMI) in patients with good allograft function significantly higher than in patients with impaired allograft function cRBF correlated with GFR
*Glomerulonephritis*		
Nishino T (2024) [39]	14 children with mesangial proliferative glomerulonephritis (mean age 11.9 ± 3.5 years)	R2* values (BOLD) correlated with glomerular sclerosis
Nishino T (2022) [40]	4 children with IgA vasculitis with BOLD MRI during the acute phase and the remission phase (median age 8.5 years)	R2* values of the acute phase higher than those of the remission phase but not significant
*Type 1 diabetic nephropathy*		
Wahba EN (2021) [41]	60 children (diabetes, 30; HC, 30)	DWI ADC correlated with eGFR and microalbuminuria
*Perinatal period*		
Faure A (2017) [42]	11 fetuses (between 28 and 32 weeks) suspected of posterior urethral valves	Abnormal ADC in patients with CKD and in interrupted pregnancies
Manganaro L (2009) [43]	88 fetuses (gestational age range 17–40 weeks) without CAKUT	ADC values correlated with gestational age
Liefke J (2023) [44]	64 adolescents (fetal growth restriction, 21; very preterm, 19; HC, 24)	No differences in T1 and T2* mapping between groups
*Urinary tract infection (pyelonephritis detection)*		
Aoyagi J (2018) [45]	7 children (median age: 2 months (0–4))	Sensitivity and specificity of DWI MRI in detecting pyelonephritis diagnosed on 99mTc-DMSA scintigraphy: 80% and 100%
Simren Y (2020) [46]	25 infants (median age: 1.7 months (0.7–5.5))	Good agreement between DWI and DMSA
Vivier PY (2014) [47]	39 children (mean age: 5.7 years)	Excellent agreement between DWI RMI and gadolinium-enhanced T1-weighted
*Urologic malformations*		
Beyoda MA (2019) [48]	25 children (median age: 7.1 years (0.3–22.7)) with pelvicalyceal dilation, with suspected or known ureteropelvic junction obstruction	ADC not correlated with functional magnetic resonance urography
Chehade H (2016) [49]	37 children (vesicoureteral reflux, 19; HC, 18)	R2* values significantly higher in HC than in reflux kidneys
Lin F (2014) [50]	76 infants (infants with unilateral hydronephrosis divided into four groups according to degree of CKD, 46; HC, 30)	Moderate positive correlation between ADCs and GFR
^**23**^**Na MRI**		
Salerno FR (2023) [51]	36 children (CKD, 19; HC, 17)	Reduced whole-leg [Na +] Z-scores: tubular disorders Elevated whole-leg [Na +] Z-score: glomerular disease and atypical hemolytic-uremic syndrome
Filler G (2020) [52]	3-year-old female with idiopathic Fanconi syndrome treated by indomethacin	Sodium decreases in skin and muscle

*ADC*, apparent diffusion coefficient; *ASL*, arterial spin labeling, *BOLD*, blood oxygen level-dependent; *CAKUT*, congenital anomalies of the kidney and urinary tract; *CKD*, chronic kidney disease; *cRBF*, cortical renal blood flow; *DWI*, diffusion weighted imaging; *eGFR*, estimated glomerular filtration rate; *HC*, healthy controls; *MRI*, magnetic resonance imaging; *SCr*, serum creatinine

In a population including 63 pediatric CKD patients, BOLD and DWI MRI parameters were found to be significantly associated with eGFR, Cystatin C, and serum creatinine levels [35]. A prospective study involving 12 pediatric and young adult CKD patients and 20 healthy control subjects revealed significant differences in kidney T1 relaxation and ADC measurements. These variations were correlated with eGFR, Cystatin C, and urinary protein-to-creatinine ratio, but also histologic cortical inflammation [36]. Another study involving pediatric patients with CKD demonstrated similar results using BOLD MRI [37].

Significant differences in DWI ADC values were observed 6 years post-transplantation when comparing eighteen kidney-transplanted adolescents with or without good allograft function (GFR ≥ or < 60 mL/min/1.73 m^2^). These differences were correlated with eGFR [38]. ASL has been employed to assess GFR and kidney blood flow in eight living kidney donors before and 1-year post transplantation. These findings need to be confirmed, but they could potentially serve as a biomarker to assess the future risk of CKD in donors post-kidney surgery [53].

In a study including 14 children with histologically proven mesangial proliferative glomerulonephritis, BOLD MRI results were correlated with glomerular sclerosis [39, 40]. The same team suggests using BOLD MRI as a non-invasive method to evaluate IgA nephritis activity in four pediatric patients as R2* value changes during MRI evaluations in both acute and remission phases of the disease [40].

In a study including 30 children with type 1 diabetes matched with healthy controls, ADC values were correlated with eGFR and microalbuminuria [41].

In a study involving 11 MRI on fetuses suspected of posterior urethral valves based on second-trimester sonography, abnormal ADC values were noted in patients with abnormal kidney function with a median follow-up of 5.1 years [42]. It is noteworthy that ADC values also vary with gestational age in fetuses without congenital anomalies of the kidney and urinary tract [43]. T1 and T2 mapping do not reveal any differences in adolescents with or without fetal growth restriction [44].

DWI MRI has been used for diagnosing acute pyelonephritis in children and predicting vesicoureteral reflux in children with pyelonephritis, compared to various standard techniques [45–47]. However, one may question the clinical added value of this technique in this specific indication compared to the already available diagnostic tools, given the cost, the availability of the technique, and the need for sedation. Additionally, diffusion and perfusion parameters have been described in diverse urologic pediatric populations, such as uretero-pelvic junction obstruction and vesicoureteral reflux [48–50].

## Multi-nuclear MRI

Traditional MRI is based on signal from protons (^1^H). Multi-nuclear MRI (multi-NMR) can detect other nuclei, such as ^19^F, ^23^Na, ^31^P, ^13^C, and ^15^N (ordered by decreasing sensitivity). These nuclei are less abundant than protons and require specialized equipment and techniques for detection. The application of multi-NMR has enabled the precise identification of molecular activities within living organisms (Fig. 1). This development holds particular significance for addressing various health conditions, including metabolic and kidney diseases.

### ^23^Na MRI

The physiology of sodium (Na^+^) homeostasis has long considered the kidney as the only regulator of sodium excretion [54]. However, it has become more complex over the past decade with the identification of a third compartment of a non-osmotically active tissue storage pool of Na^+^ in the skin and muscles, which serves as a buffer [54, 55]. ^23^Na MRI can be useful to measure tissue (skin and muscle) and kidney sodium (Fig. 2B, C) [56, 57]. In healthy people, skin and muscle sodium increase over the lifetime [57, 58]. A correlation between tissue sodium with blood pressure and left ventricular mass has been found [58]. Higher tissue sodium was described in CKD patients or patients under hemodialysis and peritoneal dialysis compared to controls [51, 59]. This tissue content can be modified by dialysis and its conditions [60]. Higher tissue content is associated with worse outcomes in dialyzed patients [61]. Lower skin and muscle sodium content after kidney transplantation was associated with improved outcomes [62]. Furthermore, a significant decrease of skin sodium was also described in adult patients with Bartter and Gitelman syndromes [63]. The use of ^23^Na MRI could provide direct visualization and quantification of abnormalities or disruptions in sodium corticomedullary gradient (CMG) in different regions of the kidney, which is even more interesting in salt-losing syndromes. Although the feasibility of obtaining significant measurements has already been published in healthy volunteers [64], no data on this specific population are currently available.

### ^31^P magnetic resonance imaging spectroscopy

^31^P magnetic resonance imaging spectroscopy (MRS) detects signals from phosphorus-containing metabolites, including phosphocreatine (PCr), inorganic phosphate (Pi), and 3 different molecules of adenosine triphosphate (ATP) alpha, beta, and gamma (Fig. 2D) [65].

Presently, only ^31^P-MRS can dynamically and non-invasively measure intracellular phosphate and ATP variations in vivo, applicable to both animals and humans. ^31^P-MRS could be a useful tool to investigate muscular function in systemic diseases since it allows measurement of oxidative metabolism in muscle during exercise and recovery [66]. This makes ^31^P-MRS interesting in nephrology for studying tubulopathy with kidney phosphate wasting and its impact on muscle metabolism. ^31^P-MRS has been utilized to investigate intracellular phosphate in adult X-linked hypophosphatemia patients. This technique provides data on muscle metabolism in this population, showing no modification of ATP in a resting state or after burosumab treatment [67, 68]. Additionally, this technique enables the study of phosphate kinetics in CKD and may play a role in detecting subclinical ischemia in CKD stage V and in dialyzed patients. Although sparse publications are available in adult CKD patients, Durozart et al. demonstrated in a previous study in adults that oxidative metabolism is impaired in the calf muscles of hemodialysis patients and is compensated by an increase in anaerobic glycolysis [69]. Our group also observed changes in Pi and ATP following acute kidney injury in an anephric pig model [70]. Furthermore, we were able to repeatedly measure spectra for 4 h during dialysis in an MRI environment in hemodialysis patients [71].

### Pediatric considerations

Scarce data (summarized in Table 1) on ^23^Na MRI are available in children. The largest pediatric cohort currently published reported 17 healthy children (11.5 ± 3.5 years), 19 healthy adults, and 19 children with CKD (12.0 ± 3.6 years) [51]. Results from this study are interesting. Healthy children had lower tissue sodium than healthy adults as expected, given that tissue sodium accumulation has been shown to correlate with age. However, Salerno et al. did not report differences between healthy children and CKD children. These findings are primarily attributed to the heterogeneity of CKD etiology in children. Elevated [Na +] Z-scores were observed among patients with non-hemolytic uremic syndrome glomerular disease and native kidneys, while lower [Na +] Z-scores were found in patients with inherited salt-wasting tubular disorders such as pseudohypoaldosteronism or Fanconi syndrome. In another publication, the same team compared tissue sodium evolution in a 13-year-old healthy female with Fanconi syndrome before and after the introduction of indomethacin, revealing a significant difference between the two situations [52]. However, no data have been published on dialyzed children or to evaluate CMG in children using this technique. No data has been published using ^31^P-MRS in children with tubulopathy with kidney phosphate wasting, CKD, or under dialysis.

## Perspectives and challenges

Multi-parametric and multi-nuclear MRI are innovative techniques with the potential to transform the conventional approach to assessing kidney function. They offer new insights into under-investigated aspects of kidney physiology such as kidney perfusion and medullary function, while providing a non-invasive way to assess kidney fibrosis, a key pathological event in CKD, glomerulonephritis, and transplant patients [9, 72]. Fibrosis is currently assessed through kidney biopsy, which involves the necessity of general anesthesia in young children and poses risks of bleeding complications or inadequacy [73]. These methods allow for in vivo, non-invasive, and comprehensive evaluation of the entire organ. Their utility is particularly evident in longitudinal studies and serial assessments, offering significant value, especially in pediatric populations even in the prenatal period and for monitoring inherited disorders. Moreover, they offer new perspectives on inherited tubular disorders associated with salt-losing syndromes or phosphate kidney wasting to apply personalized medicine. Furthermore, these techniques are not associated with radiation exposure and do not require exogenous contrast media, which is particularly interesting for patients at kidney risk. Due to advancements in technique performance, the image acquisition time has been significantly reduced to a brief duration per sequence making it particularly appealing for application in the pediatric population. Currently, these techniques remain confined to the research domain, delaying their translation into clinical practice. Several factors contribute to this gap, including technical and practical challenges. Standardized patient preparation and acquisition protocols are required for multi-parametric MRI [39, 74–77]. A description of various factors known to influence the results is mandatory, such as fasting and hydration status, dietary salt intake, tobacco use, iron status, use of 2 Tesla or 3 Tesla scanning devices, breathing or oxygen inhalation, current medications, and timing of the examination due to circadian variation. A harmonized approach is required for image and data acquisition, analysis, and processing, as different techniques are currently utilized [78]. Addressing comparability of measurements across different centers is essential. MRI availability in the hospital, especially of 3 Tesla scanners that provide better image resolution, can be challenging. MRI techniques in nephrology are at varying stages of clinical application. Integrating BOLD-type measurements into clinical practice is currently more straightforward than incorporating multi-nuclear MRI. Indeed, the latter requires specialized equipment, including a specific antenna and often a custom-made coil for imaging, which is not yet widely available. Additionally, multi-nuclear MRI necessitates experienced teams consisting of engineers, radiologists, and nephrologists. Cost expenditure associated with these techniques may be an obstacle, but potential positive health economic impacts linked to early kidney damage detection and disease progression mitigation could offset these costs. Scanning time has been reduced to 15 to 20 min, and multi-nuclear MRI is even easier to perform in children as only the leg needs to be scanned. However, sedation or general anesthesia with IV access may be necessary for children under 7 years old or those who are uncooperative. Utilizing MRI-compatible video goggles for distraction, creating a child-friendly MRI environment with photos, videos, or adjustable lighting, and incorporating small model MRI magnets or mock scanners can be beneficial [79]. Despite the significant value of multi-parametric and multi-nuclear MRI, several non-technical challenges arise. Establishing reference values for these various MRI techniques in healthy pediatric controls is crucial, necessitating further research, although including pediatric subjects can be challenging. MRI results are not specific to a particular biological process and can be influenced by multiple pathological conditions, such as changes in T1 due to edema, inflammation, and/or fibrosis. This variability can lead to conflicting results, making it difficult to pinpoint specific pathophysiological states and hindering the ability to draw definitive clinical conclusions in practical situations. Moreover, much of the existing literature is based on small case–control studies with low statistical power, often producing heterogeneous results due to non-representative sampling. Future studies should employ large-scale, rigorous, and robust study designs, integrating results from different MRI modalities and longitudinal data [80]. The results should be contextualized with the cellular and molecular pathophysiological mechanisms of diseases so that these advanced techniques can meaningfully contribute to precision and personalized medicine in kidney diseases.

## Conclusion

Multi-parametric and multi-nuclear MRI are amazing and non-invasive tools to translate molecular-level imaging to clinical applications allowing the development of medicine of precision for diagnosis and treatment of pediatric kidney disease.

## Supplementary Information

Below is the link to the electronic supplementary material.

Graphical abstract (PPTX 115 KB)
