# Supplementary material for: In vivo assessment of pediatric kidney function using multi-parametric and multi-nuclear functional magnetic resonance imaging: challenges, perspectives, and clinical applications
Source: Pediatr Nephrol. 2024 Nov 18;40(5):1539–48. doi: 10.1007/s00467-024-06560-w (PMC11946951; doi:10.1007/s00467-024-06560-w)
Supplement: Supplementary file 1 — Graphical abstract (PPTX 115 KB) [file 467_2024_6560_MOESM1_ESM.pptx]

## Slide 1
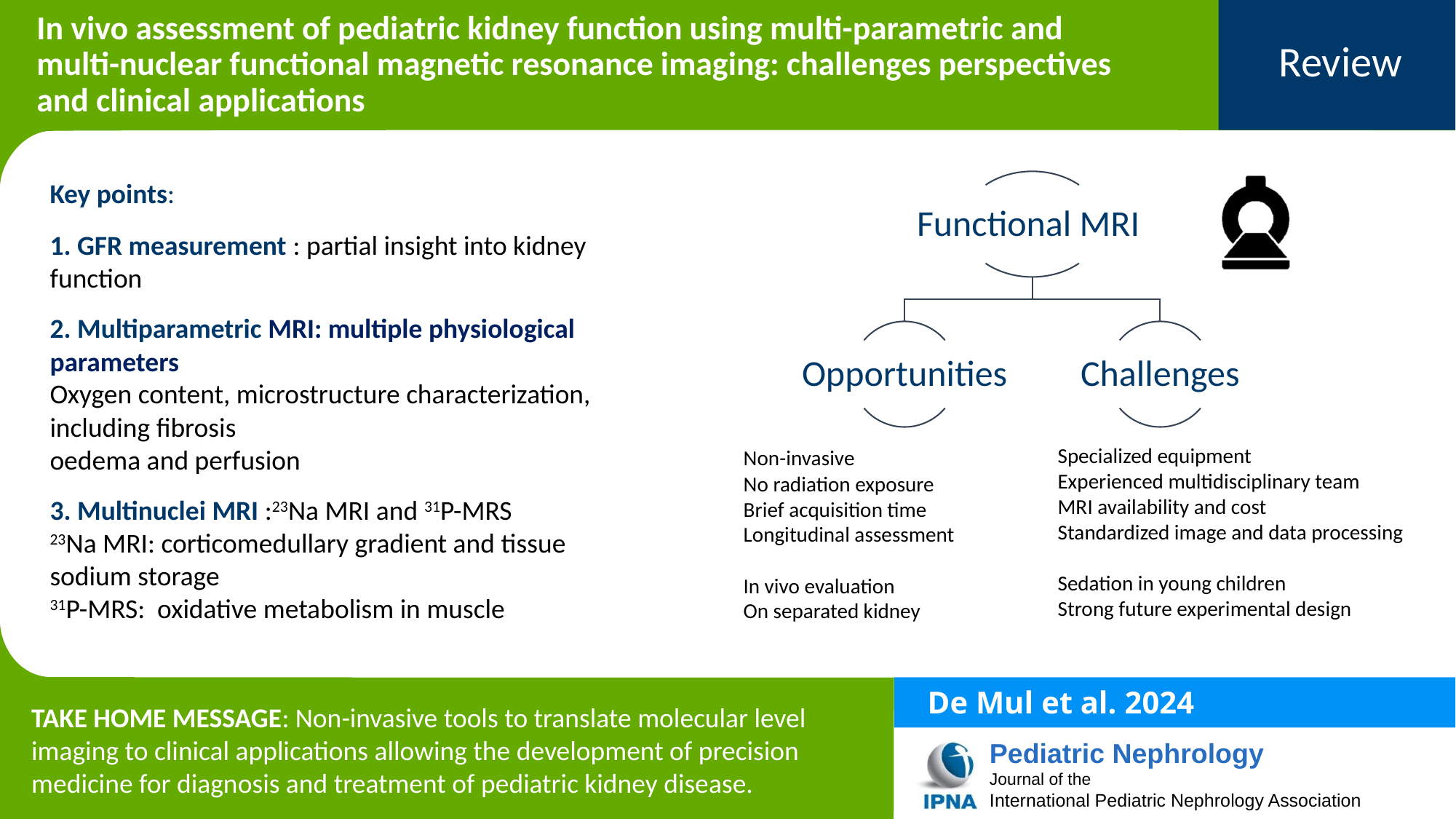

In vivo assessment of pediatric kidney function using multi-parametric and multi-nuclear functional magnetic resonance imaging: challenges perspectives and clinical applications
Key points:
1. GFR measurement : partial insight into kidney function
2. Multiparametric MRI: multiple physiological parameters
Oxygen content, microstructure characterization, including fibrosis
oedema and perfusion
3. Multinuclei MRI :23Na MRI and 31P-MRS
23Na MRI: corticomedullary gradient and tissue sodium storage
31P-MRS: oxidative metabolism in muscle
Specialized equipment
Experienced multidisciplinary team
MRI availability and cost
Standardized image and data processing
Sedation in young children
Strong future experimental design
Non-invasive
No radiation exposure
Brief acquisition time
Longitudinal assessment
In vivo evaluation
On separated kidney
De Mul et al. 2024
TAKE HOME MESSAGE: Non-invasive tools to translate molecular level imaging to clinical applications allowing the development of precision medicine for diagnosis and treatment of pediatric kidney disease.
